# Curcumin ameliorates aging-induced blood-testis barrier disruption by regulating AMPK/mTOR mediated autophagy

**DOI:** 10.1371/journal.pone.0321752

**Published:** 2025-04-24

**Authors:** Chen Xue, Zhenxing Yan, Wenjing Cheng, Dong Zhang, Rong Zhang, Hongwei Duan, Lihong Zhang, Xiaofei Ma, Junjie Hu, Jian Kang, Xiaojun Ma

**Affiliations:** 1 College of Veterinary Medicine, Gansu Agricultural University, Lanzhou, Gansu, China; 2 Key Laboratory of Animal Reproductive Physiology and Reproductive Regulation in Gansu Province, Lanzhou, Lanzhou, Gansu, China; 3 School of Animal Science and technology, Guangdong polytechnic of science and trade, Guangzhou, Guangdong, China; Shiraz University of Medical Sciences, IRAN, ISLAMIC REPUBLIC OF

## Abstract

The blood-testis barrier (BTB) is composed of tight junctions (TJ) between adjacent Sertoli cells (SCs) and is crucial for sperm growth and development. Aging-induced TJ impairment is closely related to testicular dysfunction. Curcumin, a natural compound, has been widely demonstrated to have a wide range of pharmacological activities, but its regulatory effects on tight junction damage in the testis remain unclear. We here explored the effect of curcumin on TJ function and its underlying molecular mechanism by using D-galactose (D-gal)-induced mouse testis and mouse testicular SCs (TM4) aging models *in vitro*. In this study, D-gal increased the expression of aging-related proteins p16 and p21, whereas significantly decreased the expression of TJ proteins (ZO-1, Claudin-4, Claudin-7, and Occludin). In addition, curcumin restored the adverse effects of D-gal in the SCs. Autophagy is a degradation system for maintaining cell renewal and homeostasis. D-gal significantly decreased the autophagy level, whereas curcumin restored the effect of D-gal. Using chloroquine (CQ), an inhibitor of autophagy, and rapamycin (RAPA), an activator of autophagy, it was demonstrated that autophagy plays a key role in curcumin amelioration of TJ injury in testicular SCs. Further studies unveiled that autophagy activation was mediated through the AMPK/mTOR pathway. In conclusion, curcumin ameliorates aging-induced TJ damage through AMPK/mTOR signaling pathway-regulated autophagy. This study thus clearly identifies a novel action mechanism of curcumin in the treatment of age-related male reproductive disorders.

## Introduction

The testis as a male reproductive organ have the role of producing sperm, maintaining male reproductive function and secreting androgens to regulate spermatogenesis [1]. Studies have found that aging, as a complex progressive degradation process [2], severely impairs male function and reproductive health, including testicular immune function, spermatogenesis and sperm function. Being distinct somatic cells present in seminiferous tubules, Sertoli cells (SCs) can establish the blood–testis barrier (BTB) by forming a tight junction (TJ) with neighboring SCs. SCs are directly associated with spermatogenic cells [3]. Consequently, germ cells grow in a unique protective environment. TJ is a substantial part of the BTB and is essential for preserving the BTB integrity and its functionality [4]. According to recent studies, aging can decline SCs function and induce TJ structural damage, obstructing testicular sperm production function [5].Consequently, dysfunctional production of testicular sperm can be prevented through the restoration and enhancement of SCs TJ function.

Autophagy is an intracellular degradation system in which cytoplasmic materials are transported to lysosomes for degradation [6].However, clearing the material is not the only function of autophagy, but being a dynamic cycle system, it generates new building blocks and energy for cell repair and a stable internal environment [7]. The dysfunction of autophagy with aging, as well as the reliance on autophagy for many age-protective interventions confirm the central role of autophagy in aging. Loss of autophagy function in a mice model led to cardiac dysfunction. By contrast, stimulation of autophagy function improved cardiac function and reduced heart age-related pathology [8,9]. Autophagy is required for many mechanisms regulating lifespan extension. Thus, autophagy activation may be a crucial process for alleviating TJ injury and restoring testicular function.

Adenosine monophosphate-activated protein kinase (AMPK) is an evolutionarily conserved enzyme serving as a critical sensor of the cell’s energy and nutritional conditions [10]. The mammalian target of rapamycin (mTOR) is a mechanistic target of rapamycin (RAPA) at the confluence of anabolic and catabolic metabolism [11]. It promotes cell growth by stimulating biosynthetic pathways and inhibits catabolism by decreasing cellular autophagy. AMPK mediates autophagy through multiple pathways but depends largely on mTOR downregulation [12]. AMPK regulates and extends lifespan through an integrated signaling network [13]. Unfortunately, aging can erode this valuable pathway. Aging impairs AMPK activation in rat skeletal muscles, and AMPK deficiency exacerbates aging-induced myocardial dysfunction [14]. Additionally, substantial evidence available indicates that mTOR is a negative regulator of lifespan. Lifespan extension can be achieved through the overexpression of mTOR repressors [15]. mTOR knockdown through genetic manipulation can extend lifespan in model animals [16]. Consequently, the AMPK-governed pathway exerts a major regulatory influence on autophagy and aging processes. Thus, activation of the AMPK/mTOR signaling pathway may be critical for the treatment of BTB injury due to aging.

Curcumin, an active component of the rhizomata of *Curcuma longa* Linn, is widely used as a natural medicine [17]. It has antioxidant [18], anti-inflammatory [19], antiapoptosis [20], and antibacterial functions [21]. Several clinical investigations have recently revealed that curcumin has therapeutic properties benefiting various chronic conditions related to the heart [22], lung [23], brain [24], blood vessels [25], and metabolic disorders [26]. Curcumin can restore leptin-induced TJ dysfunction in intestinal Caco-2 BBe cells [27] by increasing the expression of TJ proteins, repairing inflammation-induced impairment of the blood-milk barrier function [28], enhancing alveolar epithelial integrity under hypoxic conditions [29], increasing the diabetes-induced blood-retinal barrier capacity [30], and improving the barrier function of the blood-brain barrier under ischemic conditions [31]. And curcumin has been shown to have multiple beneficial effects on the reproductive system. curcumin can compensate for the adverse effects of testicular ischemia and improve sperm chromatin quality in mice [32]; it can restore antioxidant potential to reduce acrylamide-induced ovotoxicity in female mice [33]; it can improve body weight, glycemic control, and lipid levels in females with polycystic ovary syndrome [34]; and curcumin nanoparticles can improve semen parameters in infertile males, reduce oxidative stress and inflammatory markers, and modulate reproductive hormone levels [35]. However, the mechanism underlying the protective effect of curcumin on aging-induced male reproductive dysfunction remains unelucidated. Moreover, curcumin is a natural autophagy regulator [36]. The effect on autophagy depends on the stress-generating stimulus and the cellular environment. This effect can improve atherosclerosis, attenuate hepatic fibrosis [37], and restore neuroprotection by activating autophagy [38], and restore oxidative damage to the ovary in mice by inhibiting autophagy [39]. Thus, curcumin may act as a targeted activator of autophagy to restore aging-induced BTB damage.

Given that curcumin’s intervention on TJ function in SCs may be associated with the restoration of aging-induced BTB dysfunction. This study aimed to investigate the effect of curcumin on D-gal-induced aging, which leads to tight junction dysfunction in mouse testicular Sertoli cells, and to elucidate the significant role of autophagy in this process, given curcumin’s potential to restore aging-induced blood-testis barrier dysfunction. Our research may provide new insights into how curcumin’s protective qualities can counteract the drop in male fertility brought on by aging.

## Materials and methods

### Animals and ethics

Young male Kunming mice (age: 6–8 weeks; weight: 28–32 g) were sourced from the Laboratory Animal Center at the Lanzhou Institute of Veterinary Medicine, Chinese Academy of Agricultural Sciences (SCXK (GAN) 2020–0002). The mice were accommodated in the animal facility of the College of Veterinary Medicine at Gansu Agricultural University. These animals received humane care according to the Guidelines for the Management and Use of Laboratory Animals (Ministry of Science and Technology of China, 2006). The study was conducted following the “Guiding Principles for the Care and Use of Animals” set by the Chinese Physiological Society and was ap-proved by the Animal Care Professional Committee of Gansu Agricultural University (NO. GSAU-ETH-VMC2020–003). Before the experiments were conducted, the mice were allowed to acclimate for 1 week to a standard housing environment characterized by a 12-h light/dark cycle, a stable temperature of 23°C ± 1°C, a relative humidity of 45% ± 5%, and unlimited access to purified water and standard rodent chow.

The mice were randomly assigned to four groups: control, curcumin (purity ≥ 98.16%, Med Chem Express, New Jersey, USA) + D-gal treatment, rapamycin (RAPA, an autophagy activator, Med Chem Express) + D-gal treatment, and D-gal (purity ≥ 99.0%, Solarbio, Beijing, China) treatment, with each group containing 10 individuals. The D-gal group was subcutaneously injected with 200 mg/kg/day D-gal [40] for 60 days [41]. Concurrently, the control group administered an equal volume of saline solutions. The curcumin+D-gal group administered 200 mg/kg/day curcumin [40] at 12 h after the daily D-gal injections. The RAPA+D-gal group administered 2 mg/kg RAPA [42] at 12 h after the daily D-gal injections. All mice were euthanized by intraperitoneal injection of an overdose of sodium pentobarbital (150 mg/kg) [40] 12 h after the last treatment, followed by rapid removal of the target tissue for subsequent analysis. During the experiment, the behavioral and physiological state of the mice was closely monitored, and the mice were euthanized early if there were signs of severe distress (e.g., decreased activity, loss of appetite). Testicular tissues were harvested from the mice at postmortem. The left testes were immediately preserved at -80°C for subsequent biochemical assays. The right testis was fixed and used for histological examination (H & E), immunofluorescence (IF) studies, and transmission electron microscopy (TEM) analysis.

### Cell culture and treatment

The mouse testicular SCs cell line (TM4) was purchased from Pricella (Wuhan, China), was used in this study. The cells were grown in a DMEM/F12 medium (Gibco, Grand Island, USA), supplemented with 10% fetal bovine serum (Gibco), and further enriched with antibiotics, including 100 IU/mL penicillin (Solarbio), and 100 IU/mL streptomycin (Solarbio). The SCs were maintained in a cell culture incubator at 37°C under a 5% CO_2_ atmosphere.

Before the experiment was initiated, the cells were pretreated with a serum-free DMEM/F12 medium for 12 h to prepare them for the subsequent procedures. Various final concentrations of D-gal (5, 10, 20, 40, or 80 mg/mL) were added to the cells to simulate aging effects, along with 5, 10, 20, or 40 μM curcumin [40]. The cells were then exposed to these substances for 48 h. Furthermore, to further investigate the cellular responses, the cells were preincubated with 50 µM chloroquine [43] (CQ, an autophagy inhibitor, Med Chem Express), and 10 µM compound C [40] (CC, an AMPK inhibitor, Med Chem Express), for 1 h before the main treatments were applied.

### Western blotting

The acquired tissues and cells tissues were washed in ice-cold phosphate buffered saline (PBS) and processed using the ice-cold radioimmunoprecipitation assay (Solarbio) lysis buffer complemented with 1 mM Methyl phenyl sulfone fluoride (Solarbio), followed by western blotting. Briefly, samples containing the same amount of protein were subjected to SDS-PAGE and then transferred to a 0.45μm PVDF membrane (Millipore, MA, USA). The membranes were blocked in Tris-buffered saline containing 5% BSA for 2 h at 25 °C. The membranes were then incubated overnight at 4 °C with specific primary antibodies: P-AMPK alphaThr172 (2535, 1:1000, Cell Signaling Technology, Danvers, Massachusetts, USA), AMPK alpha (5831s, 1:1000, Cell Signaling Technology), LC3B (ab192890, 1:2000, Abcam, Cambridge, MA, USA), Beclin1 (bs-1353R, 1:500, Bioss), P-mTOR (T56571, 1:1000, Abmart, Shanghai, China), mTOR (T55306, 1:1000,Abmart), p21 (T55543S, 1:1000, Abmart), CDKN2A (TN23895S, 1:1000, Ab-mart), Occludin (27260–1-AP, 1:1000, Proteintech, Wuhan, China), Claudin-4 (16195–1-AP, 1:1000, Proteintech), Claudin-7 (29795–1-AP, 1:1000, Proteintech), ZO-1 (21773–1-AP, 1:1000, Proteintech), β-Actin (bs-0061R, 1:3000, Bioss). Next, the membranes were washed and incubated with HRP-conjugated Goat Anti-Rabbit IgG (Proteintech, SA00001–2, 1:5000)/ HRP-conjugated Goat Anti-Mouse IgG (Proteintech, SA00001–1, 1:5000) for 1 h at 37 °C. The blot was visualized by adding the ECL Plus Western blotting detection regent (Vazyme, Nanjing, China). Images were acquired using the Amersham Imager 600 chemiluminescence (GE Healthcare BioSciences AB, Sweden). Densitometry was performed using Image J software.

### Immunofluorescence analysis

The expressions of LC3, Occludin, ZO-1, Claudin-4, Claudin-7, and p21 proteins in the mouse testicular tissue and SCs were examined. Mouse tests were sectioned into 4-micron slices, deparaffinized, hydrated, and subjected to antigen retrieval. The SCs were fixed with 4% paraformaldehyde, rinsed with cold PBS, and permeabilized with 0.1% Triton x-100 for 30 min. Both tissue sections and cells were blocked with 5% BSA for 30 min and incubated overnight at 4°C with rabbit polyclonal antibodies specific to the proteins of interest. The primary antibodies were treated with Fluorescein (FITC)–conjugated Goat Anti-Mouse IgG (SA00003–1, 1:600, Proteintech)/ Fluorescein (FITC)–conjugated Goat Anti-Rabbit IgG (SA00003–2, 1:600, Proteintech) and incubated at 37°C for 45 min. The nucleus was counterstained with 1 mg/mL 4’,6-diamidino-2-phenylindole (DAPI, Solarbio). Digital images of the fluorescently labeled samples were captured using the DP73 light microscope.

### Cell viability assay

The viability of SCs was evaluated through the Cell Counting Kit-8 (CCK-8, IV08–100T, Invigentech, California, USA) assay. This assay relies on cellular dehydrogenases-induced reduction of the tetrazolium salt WST-8 by in living cells to produce a yellow, water-soluble formazan dye. The resulting color intensity correlates with the cell count, thereby indicating cell viability. The SCs were plated on 96-well plates and cultured until they reached approximately 90% confluence over 2 days. Once the treatments were applied, 10 μL CCK-8 reagent was added to each well containing 100 μL medium. The plates were incubated in the dark at 37°C for 2 h. Absorbance at 450 nm was measured to quantify the extent of formazan dye formation, thus assessing cell viability.

### Transmission electron microscopy

The tissue sections were subjected to several preparatory steps for electron microscopy. Initially, they were treated with a 3% glutaraldehyde solution and exposed to 1% osmium tetroxide solution. The sections were then subjected to tandem dehydration by using acetone. For further processing, the samples were immersed in an epoxy resin, EPOX 812, for an extended duration to allow proper infiltration and embedding. The semi-thin sections were stained with methylene blue to visualize the cellular structures. For higher-resolution imaging, ultrathin sections were cut with a diamond knife and stained with a combination of uranyl acetate and lead citrate. Finally, the prepared sections were visualized under a JEM-1400-FSASH transmission electron microscope to observe the cellular ultrastructure.

### Visualization of autophagosome formation

Autophagic vesicle formation in SCs was detected using a mCherry-GFP-LC3B-carrying adenovirus (Beyotime, Shanghai, China) vector. To summarize, SCs were grown on 6-well plates containing 10 mm coverslips. The cells were infected with the Ad-mCherry-GFP-LC3B adenovirus at 80% confluence. After the treatment was completed, the cell nuclei were stained with DAPI at 1 µg/mL for a minute. The intracellular fluorescence of Ad-mCherry-GFP-LC3B was visualized under a fluorescence microscope (Apexbio, Revolve Omega, China). This experimental procedure was performed in triplicate to ensure the results were reliable.

### Histological analysis

Mouse testicular tissues, preserved in paraffin, were sectioned into 4-μm-thick slices and stained with hematoxylin and eosin (H&E). The stained slides were examined under an Olympus-DP73 (Tokyo, Japan) light microscope to study the morphological dynamics within the testicular tissue.

### Statistical analysis

Statistical evaluations were conducted using SPSS software (version 22.0, SPSS Inc., Chicago, IL, USA). The normality and homoscedasticity of all data were examined. One-way analysis of variance and Duncan’s multiple range test were subsequently implemented to determine significant differences in data. The mean ± SEM was adopted to present quantitative data. *P<*0.05 was established as the threshold for statistical significance.

## Results

### D-gal-induced aging led to TJ damage in SCs *in vitro*

**Fig 1:**
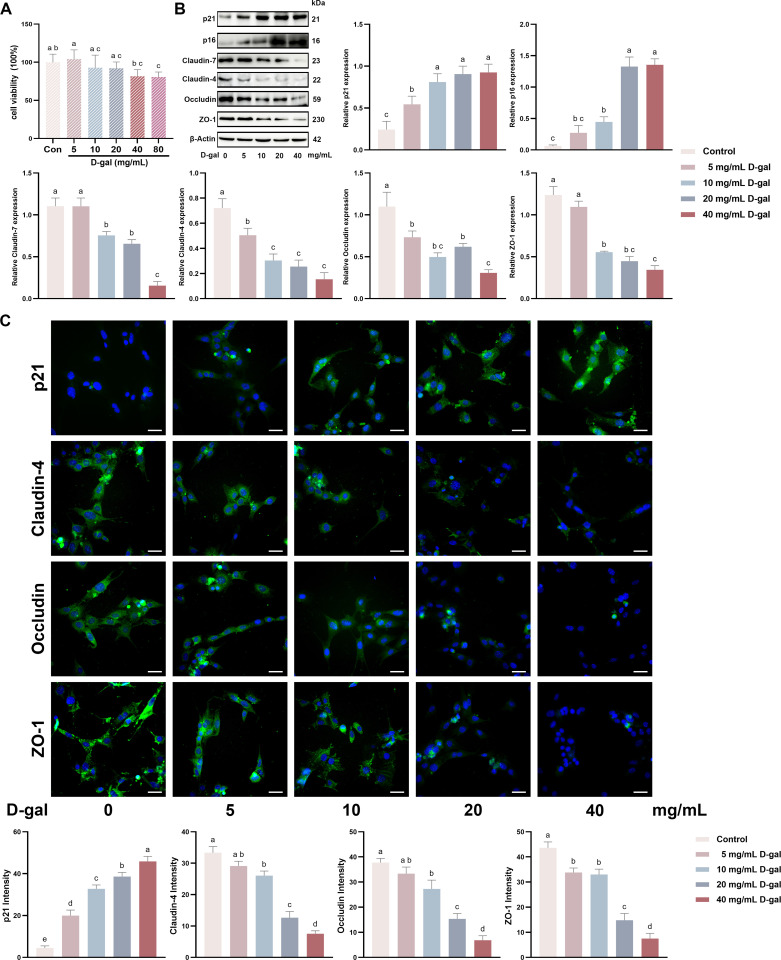
D-gal-induced aging led to tight junction damage in SCs *in vitro.* (A) The viability of SCs was evaluated after exposure to D-gal at concentrations of 0, 5, 10, 20, 40, and 80 mg/mL. (B) Following a 48-h treatment with D-gal concentrations of 0, 5, 10, 20, and 40 mg/mL, Western blotting was performed to assess the expressions of Claudin-4, Claudin-7, p21, ZO-1, p16, and Occludin proteins in SCs. (C) Cellular immunofluorescence (scale bar = 50 μm) was utilized to examine and quantify the expressions of p21, Claudin-4, Occludin, and ZO-1 in SCs treated with varying D-gal concentrations (0, 5, 10, 20, and 40 mg/mL). Data is presented as the mean ±standard error of the mean (SEM). The data are statistically significant between different letters(*p*<0.05).

To determine whether aging affects the BTB function by disrupting SCs TJ, a standard mouse testicular SCs line (TM4) was cultivated *in vitro*. D-gal induces aging-like effects that parallel those of natural aging [44]; hence, D-gal-treated SCs were selected to simulate aging in this study. The viability of the SCs was assessed after a 48-h treatment with various D-gal concentrations. D-gal concentrations at 0–20 mg/mL caused no significant alterations in cell viability compared with the control group. However, higher D-gal concentrations (40 and 80 mg/mL) markedly decreased cell viability (Fig 1A). Subsequent treatment of the SCs with different D-gal concentrations for 48 h and western blotting unveiled that the expression of aging-associated proteins, p16 and p21, were sustainably elevated in the treated cells, suggesting that D-gal treatment mimicked SCs aging. Compared with the control group, 0–5 mg/mL D-gal exerted no significant effect on the expression of Claudin-7 and ZO-1. By contrast, 10–40 mg/mL D-gal significantly reduced the expression of the TJ proteins (Fig 1B). Cellular immunofluorescence assays indicated that varying D-gal concentrations consistently increased the expressions of the aging-associated protein p21 compared with the control group. D-gal dose-dependently reduced the expression of the TJ proteins in SCs (Fig 1C). Taken together, these data demonstrate that D-gal treatment induces aging-like effects in SCs by reducing TJ protein expression.

### Curcumin restores D-gal-induced SCs TJ damage *in vitro*

**Fig 2:**
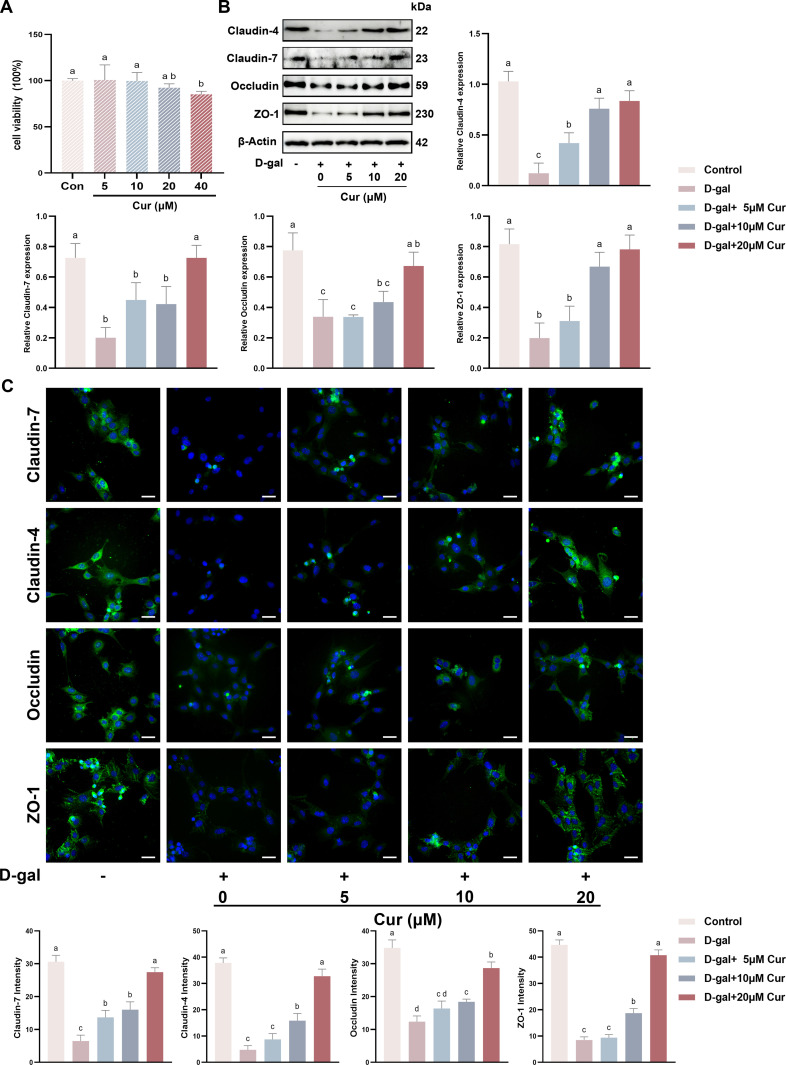
Curcumin restores D-gal-induced SCs TJ damage *in vitro.* (A) The survival rate of SCs was determined after exposure to curcumin at concentrations of 0, 5, 10, 20, and 40 μM. (B) SCs were incubated with curcumin at concentrations of 0, 5, 10, and 20 μM in the presence of 40 mg/mL D-gal for 48 h, followed by Western blotting analysis to evaluate the expressions of Claudin-4, Claudin-7, ZO-1, and Occludin proteins. (C) A cellular immunofluorescence assay (scale bar = 50 μm) was performed to assess and quantify the expressions of Claudin-4, Claudin-7, Occludin, and ZO-1 in SCs co-treated with curcumin at concentrations of 0, 5, 10, and 20 μM and 40 mg/mL D-gal. Data is presented as the mean ±standard error of the mean (SEM). The data are statistically significant between different letters(*p*<0.05).

Subsequently, the study examined the protective role of curcumin against D-gal-induced aging and its potential to ameliorate the disruption of TJ functions in SCs. The viability of SCs was assessed after incubation with various curcumin concentrationsfor 48 h. Within this concentration range, 0–20 μM curcumin had no significant impact on cell viability and 40 μM curcumin significantly reduced cell viability compared to the control group (Fig 2A). The SCs were then treated with varying curcumin concentrations alongside D-gal for 48 h. Western blotting revealed that curcumin dose-dependently restored D-gal-stimulated TJ protein expression (Fig 2B). Consistent with these findings, cellular immunofluorescence assays revealed that curcumin dose-dependently increased the expressions of the TJ proteins in the D-gal-treated SCs (Fig 2C). Taken together, curcumin can restore the functional impairment of SCs TJ due to D-gal-induced aging *in vitro*.

### D-gal-induced aging leads to reduced autophagy levels in SCs, which are restored by curcumin *in vitro*

**Fig 3:**
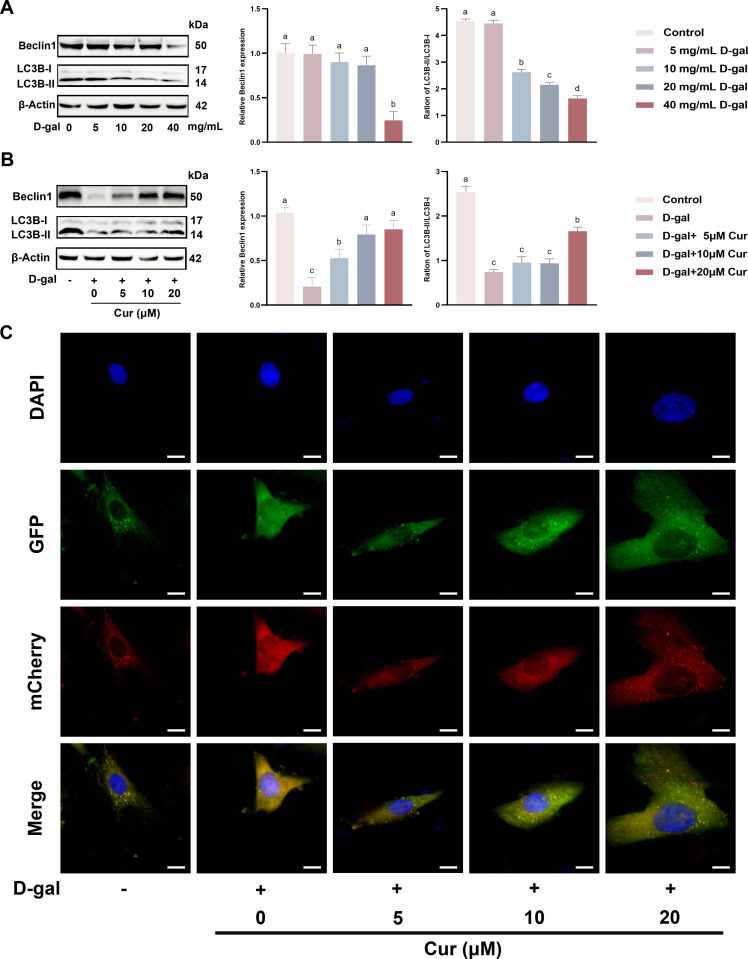
D-gal-induced aging leads to reduced autophagy levels in SCs, which are restored by curcumin *in vitro.* (A) Protein expression and quantification of Beclin1 and LC3 in SCs treated with different concentrations of D-gal (0, 5, 10, 20, 40 mg/mL) by Western blotting. (B) The expression and quantification of Beclin1 and LC3 proteins in SCs were evaluated after co-treatment with a range of curcumin concentrations (0, 5, 10, and 20 μM) along with a constant 40 mg/mL D-gal by using Western blotting. (C) The formation of autophagosomes was visualized in SCs following their co-treatment with the different series of curcumin concentrations (0, 5, 10, and 20 μM) in the presence of 40 mg/mL D-gal. Data is presented as the mean ±standard error of the mean (SEM). The data are statistically significant between different letters (*p*<0.05).

Autophagy is a major intracellular degradation system involved in cellular repair through dynamic cycling to maintain the stability of the internal environment [6]. Autophagy dysfunction may be induced by aging. Many geroprotective interventions depend on autophagy [7,9]. Therefore, curcumin was tested for its role in restoring autophagy levels in D-gal-induced SCs TJ injury *in vitro*. SCs were treated with various D-gal concentrations for 48 h. According to western blotting results, 0–20 mg/mL D-gal caused no significant alterations in Beclin1 expressions. By contrast, 40 mg/mL D-gal significantly decreased Beclin1 expression (Fig 3A). Exposure to varying D-gal concentrations consistently reduced the LC3 protein expressions in the cells (Fig 3A), suggesting that aging induces a lack of autophagy, thus causing autophagy dysfunction. Subsequently, SCs were then concurrently treated with curcumin along with D-gal for 48 h. Western blotting unveiled that curcumin dose-dependently restored Beclin1 and LC3 expression in D-gal-stimulated SCs (Fig 3B). In addition, consistent with the Western blotting results, the Ad-mCherry-GFP-LC3B results indicated that D-gal-induced aging inhibited autophagosome formation, which was effectively restored by curcumin (Fig 3C). In summary, curcumin restored D-gal-induced decrease in the autophagy level of SCs.

### Curcumin restores D-gal-induced SCs TJ injury through autophagy activation *in vitro*

**Fig 4:**
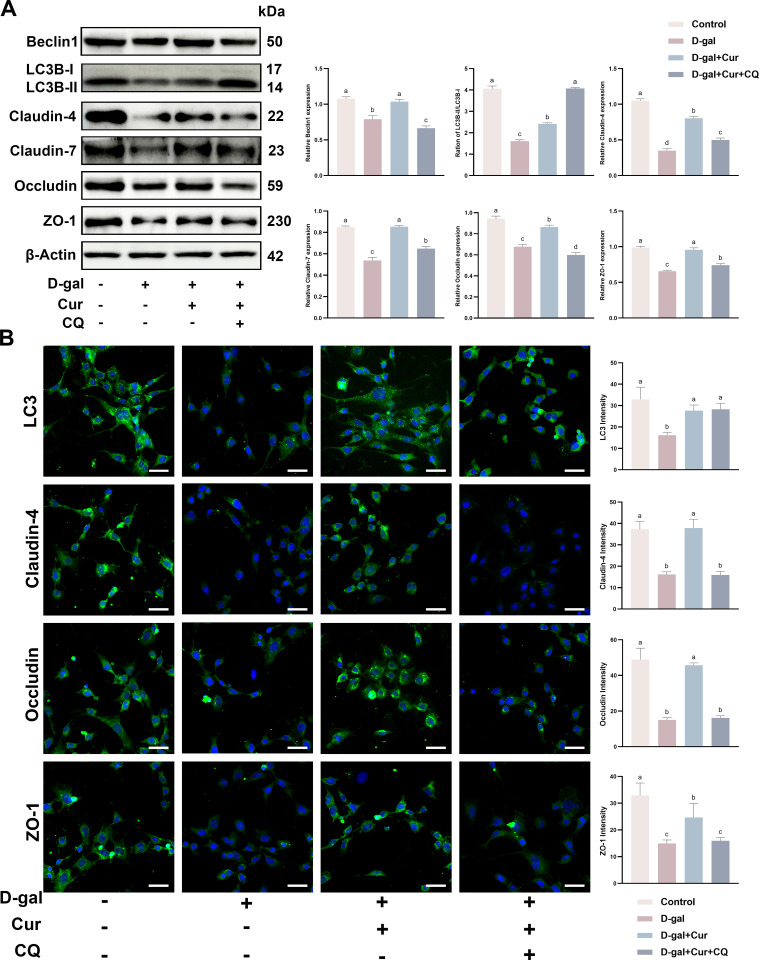
Curcumin restores D-gal-induced SCs TJ injury through autophagy activation *in vitro.* (A) Protein expression and quantification of Beclin1, LC3, Clauin-4, Claudin-7, Occludin and ZO-1 in SCs after D-gal (40 mg/mL) and curcumin (20 µM)/CQ (50 µM) treatment by Western blotting. (B) Cellular immunofluorescence assay for protein expression and quantification of LC3, Claudin-4, Occludin, and ZO-1 in SCs after treatment with D-gal (40 mg/mL) and curcumin (20 µM)/CQ (50 µM). Data is presented as the mean ±standard error of the mean (SEM). The data are statistically significant between different letters(*p*<0.05).

We further investigated the mechanistic role of curcumin in restoring D-gal-induced SCs TJ injury *in vitro*. To test whether the role of curcumin in restoring D-gal-induced SCs TJ injury *in vitro* is mediated through the autophagy restoration pathway, the autophagy inhibitor chloroquine (CQ) was added to the SCs. As expected, curcumin restored the expression of proteins involved in D-gal-induced TJ damage and autophagy *in vitro*. By contrast, CQ addition significantly inhibited the expression of all these proteins, except LC3 (Fig 4A). Similar to the western blotting, cellular immunofluorescence unveiled that curcumin restored the expression of proteins associated with D-gal-induced TJ damage and autophagy *in vitro*. By contrast, CQ addition significantly suppressed the expression of all TJ proteins except LC3 (Fig 4B). These results confirm that curcumin restores D-gal-induced SCs TJ damage by activating autophagy *in vitro*.

### Curcumin restores aging-induced disruption of the BTB in mice by activating autophagy in mouse testes injected with D-gal

**Fig 5:**
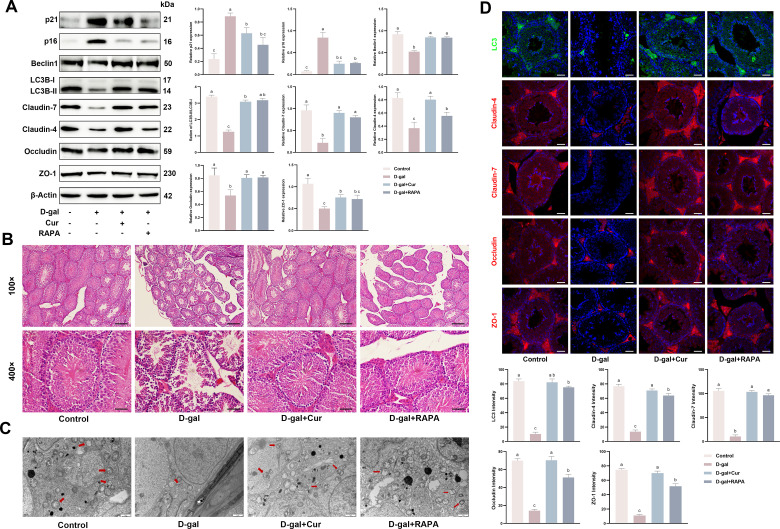
Curcumin restores aging-induced disruption of the BTB in mice by activating autophagy in mouse testes injected with D-gal. The mice were assigned to the control group (administered 200 mg/kg saline), model group (administered 200 mg/kg D-gal), curcumin-treated group (administered 200 mg/kg curcumin + 200 mg/kg D-gal), and rapamycin group (administered 200 mg/kg D-gal + 2 mg/kg RAPA). (A) Western blotting was utilized to detect and quantify the expressions of proteins including p21, p16, Beclin1, LC3, Claudin-4, Claudin-7, Occludin, and ZO-1. (B) Hematoxylin and eosin (H&E) staining was conducted to examine the histological alterations within the testes. The upper panels display the modifications in the seminiferous tubules at a 100× magnification (scale bar = 200 μm), while the lower panels present a closer view at a 400× magnification (scale bar = 50 μm). (C) Transmission electron microscopy (TEM) was employed to visualize autophagic structures in the mouse testis (scale bar = 1 μm), arrows indicate autophagosomes. (D) Tissue immunofluorescence for protein expression and quantification of LC3, Claudin-4, Claudin-7, Occludin, and ZO-1. Data is presented as the mean ±standard error of the mean (SEM). The data are statistically significant between different letters (*p*<0.05).

Subsequently, the effect of curcumin on the disrupted BTB in the testes of mice chronically injected with D-gal was evaluated. H&E staining indicated that the mice receiving long-term D-gal injections experienced marked distortion and damage to the shape and structure of the testicular tubules, and a diminished spermatocyte layer count. By contrast, when curcumin or the autophagy-promoting agent RAPA was ad-ministered, the tubular morphology and structure exhibited a significant recovery, along with an increase in the number of spermatocyte layers (Fig 5B). Furthermore, TEM revealed a reduction in the number of double-membraned vesicular structures within the D-gal-treated group. This decrease was notably reversed by curcumin and RAPA treatments, thereby increasing the presence of these vesicular structures (Fig 5C). Western blotting data corroborated the experimental findings *in vitro*, demonstrating that D-gal treatment increased the expression of testicular aging-related proteins in mice. Concurrently, the expression of proteins associated with TJ and autophagy decreased. The expressions of these proteins were restored following curcumin or rapamycin treatment (Fig 5A). Tissue immunofluorescence results were similar to western blotting results, in that the expression of proteins associated with TJ and autophagy decreased in the D-gal-treated mouse testes, which were restored following curcumin or rapamycin treatment (Fig 5D). In conclusion, the *in vivo* data suggest that curcumin attenuates testicular BTB damage by activating autophagy and thus promoting TJ function restoration in SCs.

### Activation of the AMPK/mTOR signaling pathway correlates with curcumin-mediated autophagy activation to restore D-gal-induced SCs TJ damage

**Fig 6:**
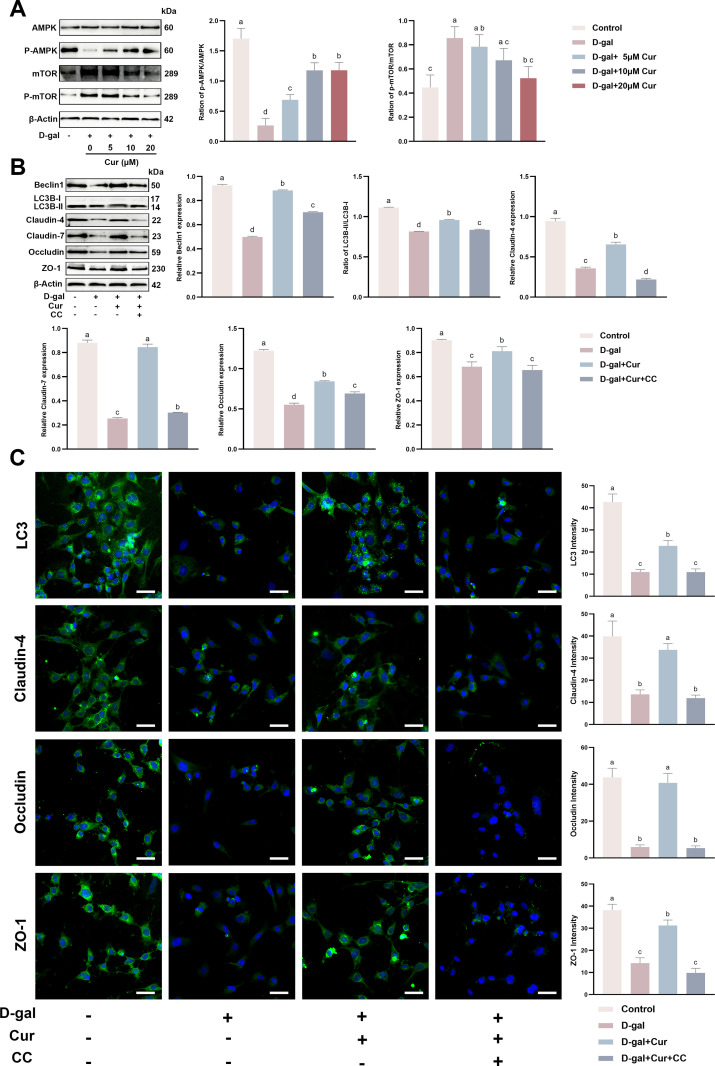
Activation of the AMPK/mTOR signaling pathway correlates with curcumin-activated autophagy to restore D-gal-induced tight junction damage in SCs. (A) Protein expression and quantification of p -AMPK/AMPK and p-mTOR/mTOR after co-treatment of SCs with different concentrations of curcumin (0, 5, 10, and 20 μM) and 40 mg/mL D-gal by Western blotting. (B) Protein expression and quantification of Beclin1, LC3, Claudin-4, Claudin-7, Occludin, and ZO-1 in SCs after treatment with D-gal (40 mg/mL) and curcumin (20 µM)/CC (10 µM) by Western blotting. (C) Cellular immunofluorescence assay (scale bar = 50 μm) for protein expression and quantification of Beclin1, LC3, Claudin-4, Claudin-7, Occludin, and ZO-1 in SCs after D-gal (40 mg/mL) and curcumin (20 µM)/CC (10 µM) treatment. Data is presented as the mean ±standard error of the mean (SEM). The data are statistically significant between different letters (*p*<0.05).

Ultimately, the study delved into the molecular mechanisms underpinning how curcumin counteracts the D-gal-induced impairment of the SCs TJ function. The AMPK/mTOR signaling pathway is a cellular sensor for energy and signaling [45]. It oversees numerous vital cellular processes, such as autophagy [46]. Furthermore, AMPK is pivotally involved in aging. Consequently, the influence of the AMPK/mTOR signaling pathway on the protective effect of curcumin against aging-induced decline in autophagy was examined in SCs. Western blotting indicated that D-gal-induced aging significantly reduced the ratio of phosphorylated to total AMPK (p-AMPK/AMPK) and correspondingly increased the ratio of phosphorylated to total mTOR (p-mTOR/mTOR) in SCs. Moreover, curcumin dose-dependently restored these expressions (Fig 6A). To test whether the role of curcumin in restoring D-gal-induced SCs TJ damage *in vitro* by activating autophagy is mediated through the AMPK/mTOR signaling pathway, the AMPK inhibitor Compound C was added to SCs. Western blotting showed that curcumin restored the expression of proteins involved in D-gal-induced TJ damage and autophagy *in vitro*. By contrast, the expression of all these proteins was significantly inhibited by the CC (Fig 6B). Similar to western blotting, cellular immunofluorescence unveiled that curcumin restored the expression of proteins involved in D-gal-induced TJ damage and autophagy *in vitro*. By contrast, the expression of all these proteins was significantly suppressed by the added CC (Fig 6C). These results confirm that curcumin-induced autophagy activation to restore D-gal-induced SCs TJ damage *in vitro* correlates with the activation of the AMPK/mTOR signaling pathway.

## Discussion

**Fig 7:**
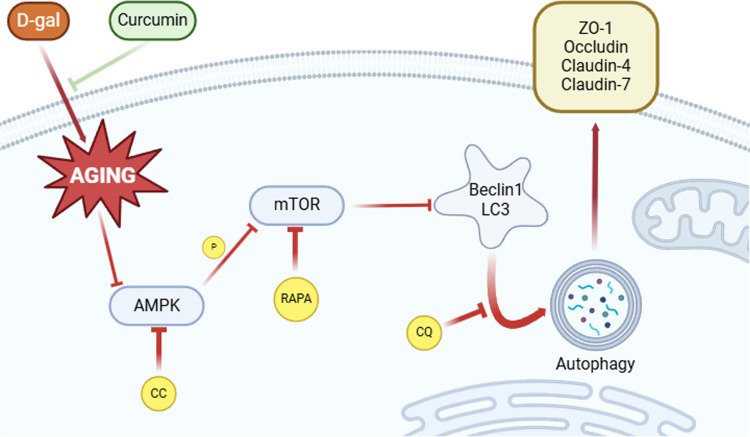
Schematic diagram of the mechanism by which curcumin ameliorates aging-induced TJ damage in SCs. Image abstract: In mouse SCs, D-gal-induced aging leads to a severe lack of cellular autophagy as well as tight junction damage. Curcumin ameliorates aging-induced TJ damage in mouse SCs by activating AMPK/mTOR-mediated autophagy.

With aging, the structure and function of the testis decline, thereby affecting the male reproductive function and sperm quantity and quality [47]. During spermatogenesis, the SCs wrapped around the germ cells provide nutrition and participate in BTB formation, a key factor affecting germ cell development and spermatogenesis [48,3]. In this study, the *in vitro* and *in vivo* data unveiled that curcumin protects aging-induced TJ damage, and the underlying mechanism is to restore autophagy dysfunction by activating the AMPK/mTOR pathway (Fig 7).

The characteristics of chronic D-gal exposure-induced premature aging are similar to those of natural aging, including shortened lifespan, oxidative stress, and in-creased mitochondrial dysfunction [33]. In the present study, SCs were treated with D-gal, thus mimicking the aging of SCs. Studies have reported that mRNA and protein expression of p21 and p16 genes is increased in senescent cells, which contributes to a state of permanent cell cycle arrest. Thus, p21 and p16 are two key regulators of the cell life cycle [49]. As anticipated, the assay outcomes for p21 and p16 unveiled that D-gal treatment effectively simulated aging in SCs. Additionally, aging can downregulate TJ protein expression and localization, thereby impairing the BTB and exacerbating spermatogenic epithelium disruption, ultimately leading to spermatogenic dysfunction or even infertility [50]. Considering the critical functions of SCs in spermatogenesis and BTB integrity construction, and the detrimental effects of aging on SC function, comprehending the mechanism underlying SC aging is crucial for prolonging the male reproductive function. The present study results showed that high D-gal concentrations significantly reduced the expression of TJ proteins, confirming that the onset of aging also disrupts the TJ of SCs. By establishing a mouse model of aging, which was consistent with the in vitro results, we further verified that D-gal-induced aging causes BTB dysfunction in mice.

As an antioxidant and antiaging natural polyphenolic compound, curcumin and its metabolites can extend the average lifespan of some model organisms of aging [51]. Accumulation of large amounts of amyloid in the brain is among the most prominent causes of Alzheimer’s disease. Curcumin improves the function of brain nerve cells by preventing their damage [52]. Moreover, curcumin exerts a therapeutic effect on skin diseases [53], cardiovascular diseases [54], diabetes [55], and testicular torsion damage [56]. In addition, curcumin reduced apoptosis and increased testosterone secretion in testicular interstitial cells of senescent mice [57]. We here explored the regulatory effect of curcumin on aging in SCs and its association with the diminished TJ function. Curcumin blocked aging-induced damage to SCs TJ, thereby restoring the BTB function. Subsequently, a mouse model of aging was established, further validating the therapeutic role of curcumin in aging-induced TJ function impairment. Curcumin is known to inhibit and reverse TJ dysfunction in the retinal pigment epithelium [30], renal fibrosis [58], blood–brain barrier [59], intestinal epithelial cells [60], alveolar epithelial cells [29], and mammary epithelial cells [8]. Consistent with the current investigation, curcumin possesses various therapeutic properties and exhibits broad efficacy in addressing age-related conditions and aging-induced TJ dysfunctions.

Autophagy is a vital and universal mechanism for degrading damaged macro-molecules and dysfunctional organelles throughout cellular processes. Thus, cellular autoregulation of autophagy directly improves protease homeostasis and somatic cell maintenance. Autophagy is a fundamental cellular process operating in healthy cells for removing and repurposing damaged proteins and organelles. However, aging-induced autophagy dysfunction causes intracellular environmental disturbances, ultimately resulting in cell death [61]. By contrast, non-cell-autonomous prolongation of the lifespan of an organism can be achieved through tissue-specific induction of autophagy [62]. Furthermore, autophagy is a crucial player in the survival and functionality of testicular SCs under normal physiological conditions as well as during pathological states. Suppressing the expression of autophagy-associated proteins in testicular SCs can decrease autophagy levels, thereby affecting the fertility of male mice. Other studies have shown that enhanced autophagy can mitigate aging-induced deterioration of TJ function at the blood–brain barrier [31,63]. In the present study, curcumin restored aging-induced autophagy dysfunction in SCs, which was later verified by establishing a mouse model of aging. Curcumin restored aging-induced TJ functional impairment and modulated aging-induced autophagy dysfunction. Subsequently, like curcumin, the autophagy activator RAPA restored aging-induced TJ injury to a certain extent, whereas the autophagy inhibitor CQ inhibited the therapeutic effect of curcumin on TJ function. These results suggest that autophagy restoration is a crucial method by which curcumin alleviates aging-induced testicular TJ injury.

AMPK is a pivotal kind of kinase with a crucial role in maintaining energy balance and is involved in various signaling pathways [64]. AMPK activation enhances mitochondrial performance, stimulates fatty acid and cholesterol production, exerts a significant regulatory effect on curbing inflammatory processes, and contributes to decelerating or halting aging, which is critical for the treatment of cardiovascular disease and other aging-related diseases [65]. The AMPK signaling network intricately connects autophagy and aging processes through its interplay with the mTOR pathway. The modulation of AMPK/mTOR-dependent autophagy safeguards cardiomyocytes and postpones their senescence. Conversely, excessive mTOR activation is associated with diminished autophagy and accelerated aging in Zmpste24-deficient mice. Additionally, the AMPK/mTOR signaling pathway is closely related to autophagy-regulated TJ function in intestinal epithelial barrier and blood-brain barrier injuries. In this study, curcumin increased the p-AMPK/AMPK ratio but decreased the p-mTOR/mTOR ratio in an in vitro model of aging. Furthermore, the AMPK inhibitor CC exerted a reversal effect on curcumin-mediated increase in autophagy levels and TJ function, which suggests that curcumin activates autophagy and thus restores aging-induced testicular TJ damage. This effect is achieved by modulating the AMPK/mTOR signaling pathway.

In summary, the available data demonstrates that curcumin restores the TJ function in SCs, an *in vitro* model of aging, and improves BTB function in a mouse model of aging. Curcumin exerts these effects through autophagy activation by targeting the AMPK/mTOR signaling pathway. The collective study findings pave the way for understanding the novel mechanism through which curcumin protects against age-related male reproductive dysfunctions, revealing that autophagy restoration by targeting the AMPK/mTOR signaling pathway may be effective for treating age-related BTB diseases.

### Implications

The effects of aging on autophagy and testis, as well as the role of curcumin in restoring autophagy and BTB, were well demonstrated in this study. A new avenue was explored for the treatment of aging-induced male reproductive disorders.
